# A Comparison of Tracheal Intubation Using Direct Laryngoscope and Video Laryngoscope in the Sellick and Trendelenburg Position with That Using Direct Laryngoscope in the Supine Sniffing Position: A Randomized Controlled Trial

**DOI:** 10.3390/jcm13154482

**Published:** 2024-07-31

**Authors:** Yun-Jeong Chae, Jung-Yoon Moon, Min-Gyu Lee, Han-Bum Joe

**Affiliations:** Department of Anesthesiology and Pain Medicine, Ajou University School of Medicine, Suwon 16499, Republic of Korea; yjchae06@hotmail.com (Y.-J.C.); jymoon1028@gmail.com (J.-Y.M.); blandy0101@naver.com (M.-G.L.)

**Keywords:** general anesthesia, tracheal intubation, pulmonary aspiration, video laryngoscope, Sellick and Trendelenburg position

## Abstract

**Background**: Tracheal intubation in the Sellick and Trendelenburg position (ST position) can prevent pulmonary aspiration but increase the difficulty of tracheal intubation. We compared tracheal intubation using video and direct laryngoscopy in the ST position with direct laryngoscopy in the supine sniffing position to evaluate the overall intubation performance. **Methods**: One hundred and twenty patients were randomly assigned to three groups: direct laryngoscope in the supine sniffing position (control), direct laryngoscope in the ST position (ST direct), and video laryngoscope in the ST position (ST video). The primary outcome was the intubation time; secondary outcomes included the first attempt success rate of tracheal intubation, intubation difficulty scale score, operator’s subjective assessment of intubation difficulty, and modified Cormack–Lehane grades. **Results**: The median intubation times were greater in the ST direct (36.0 s) and video (34.5 s) than the control (28.0 s) groups. The first attempt success rate decreased in the ST direct (77.5%) but not the video (95.0%) group compared with the control group (100%). **Conclusions**: The challenges of tracheal intubation in the ST position, aimed at reducing the risk of pulmonary aspiration, can be mitigated by using a video laryngoscope, despite slightly longer intubation times.

## 1. Introduction

Pulmonary aspiration is a serious complication that must be avoided during anesthesia induction [1]. Rapid sequence induction and intubation (RSII) is considered the standard of care for patients who are at high risk of pulmonary aspiration [2,3,4]; however, the recommended or optimal patient position during RSII remains a topic of controversy. The head-down position has the advantage of reducing possible contamination of the airway by gastric contents owing to gravity during active vomiting [2,3]. Some manikin studies have reported that a combination of the Trendelenburg position and full cervical extension (Sellick position) can prevent airway contamination attributed to refluxed gastric contents [5,6].

In the Sellick position, the cervical spine is fully extended by bending the table under the occipital region [7]. Moreover, the possibility of pulmonary aspiration by gravity can be prevented in the Sellick position with a head-down tilt of approximately 10 degrees, as the carina is positioned higher than the larynx and oral cavity [5,6]. Therefore, by using the Sellick and Trendelenburg (ST) position, tracheal intubation can be performed more safely in patients at high risk of pulmonary aspiration. However, tracheal intubation is potentially more challenging in this position than the commonly performed sniffing position [8]. Hence, there may be reluctance in actively using the ST position in clinical practice unless actual regurgitation or pulmonary aspiration occurs. Once these difficulties in tracheal intubation are overcome, the advantages of the ST position, such as preventing pulmonary aspiration or reducing exacerbations, can be actively utilized.

Recently, video laryngoscopy has been widely considered as a method of tracheal intubation; moreover, the difficulty of tracheal intubation has been greatly reduced compared with that performed with a direct laryngoscope [9,10,11]. Therefore, video laryngoscopy may facilitate tracheal intubation in the ST position in patients at high risk of pulmonary aspiration.

In this study, we aimed to compare tracheal intubation using video and direct laryngoscopy in the ST position and tracheal intubation using a direct laryngoscope in the supine sniffing position to determine the differences in intubation time, success rate, and difficulty of tracheal intubation.

## 2. Materials and Methods

This prospective randomized controlled trial was approved by the Institutional Review Board (AJOUIRB-OBS-2019-507) of our institution and registered at ClinicalTrials.gov (NCT04457453). This study was conducted at the Ajou University Hospital (Suwon, Republic of Korea) between March 2021 and April 2022. All patients were provided adequate information regarding the study, and written informed consent was obtained from all patients. This study was conducted in accordance with the CONSORT guidelines and the principles of the Declaration of Helsinki.

### 2.1. Patients

The inclusion criteria were an age of 19–70 years, an American Society of Anesthesiologists (ASA) physical status I or II, and being scheduled for general anesthesia with orotracheal intubation. The exclusion criteria were anatomical deformity of the head and neck; pulmonary aspiration risk; limited range of motion or pain in the cervical spine; risk of dental injury during tracheal intubation; and a body mass index >35 kg/m^2^.

Patients were randomly allocated to one of three groups based on the method of tracheal intubation and patient position, namely, direct laryngoscopy in the supine sniffing position (control group), direct laryngoscopy in the ST position (ST direct group), and video laryngoscopy in the ST position (ST video group), using the Excel “Random” function (Microsoft Office 2010, Microsoft Corporation, Redmond, WA, USA). A colleague who was not involved in this study conducted the allocation process. The randomization results were concealed within opaque, serially numbered envelopes. The envelope was opened after the patient was brought to the operating room to identify the corresponding group, and tracheal intubation was performed accordingly.

### 2.2. Anesthesia and Patient Evaluation

**Figure 1:**
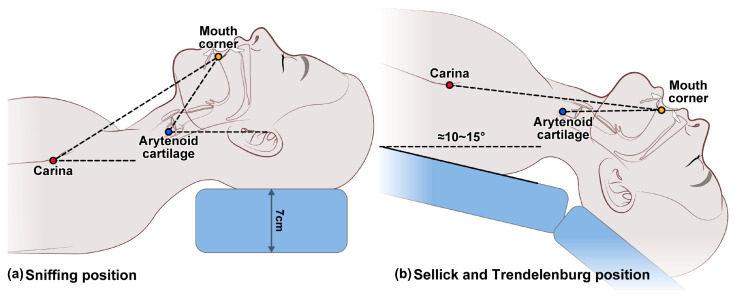
Patient positions for tracheal intubation: (**a**) supine sniffing position for the control group; (**b**) full cervical spine extension (Sellick position) and Trendelenburg position (head-down tilt, 10–15°) for the ST direct and ST video groups. The Sellick and Trendelenburg position was first set by performing full cervical extension without a pillow and then performing a head-down tilt such that the imaginary line connecting the arytenoid cartilage and the corners of the mouth was horizontal.

No premedication was administered before induction, and standard monitors were used as the patients arrived in the operating room. The patients’ blood pressure, electrocardiogram, heart rate, oxygen saturation, and end-tidal carbon dioxide levels were monitored noninvasively. For anesthesia induction, 2 mg/kg propofol and 1.5 µg/kg fentanyl were intravenously administered. After loss of consciousness, 0.6 mg/kg rocuronium was intravenously administered and manual ventilation was performed with sevoflurane for 2 min. The tracheal tube was prepared by inserting a stylet into the tube and setting it in the shape of a hockey stick by bending it at 90° just above the cuff. The inner diameters of the tracheal tube for male and female patients were 8.0 and 7.0 mm, respectively. Two minutes after rocuronium injection, sufficient neuromuscular blockade was achieved [12], and the laryngoscopic view of the patient was assessed using a direct laryngoscope in the supine sniffing position. Afterward, the patient’s position was changed according to the study group. In the control group, tracheal intubation was performed using the direct laryngoscope in the supine position with a 7 cm high pillow placed beneath the head, which was the original position (supine sniffing position, Figure 1a). In the other two groups, patients were changed to the ST position. The ST position was first set by performing full cervical extension without a pillow, followed by head-down tilting such that the imaginary line connecting the arytenoid cartilage and the corners of the mouth was horizontal (Figure 1b). The arytenoid cartilage was assumed to be located at the point of the oblique line of the thyroid cartilage, which was palpable in the posterior direction at the laryngeal prominence level. At this time, the head-down tilt angle of the operating table in the ST position was measured.

Tracheal intubation was performed by an anesthesiologist with >20 years of experience using the direct laryngoscope or video laryngoscope assigned to each group, and the laryngoscopic view seen with the corresponding laryngoscope was re-evaluated in the ST direct and ST video groups. The laryngoscopic views evaluated in this study were presented as modified Cormack–Lehane grades [13]. A Macintosh blade was used for direct laryngoscopy, and an AceScope^TM^ (Acemedical Co., Seoul, Republic of Korea) equipped with a disposable Macintosh blade was used for video laryngoscopy.

The intubation time was defined as the time from the insertion of the laryngoscope into the mouth to measure the end-tidal carbon dioxide level. If esophageal intubation was achieved on the first attempt, tracheal intubation was not achieved within 90 s, or the modified Cormack–Lehane grade was IV, the intubation attempt was stopped and recorded as a failure. If failure of tracheal intubation was recorded on the first attempt, video laryngoscopy was performed by switching the patient to the supine sniffing position. In this case, the time spent on the first failed intubation attempt was added to the intubation time of the second attempt to represent the intubation time.

The difficulty of tracheal intubation was assessed using the intubation difficulty scale (IDS) and the operator’s subjective assessment of intubation difficulty. The IDS score, which is a function of seven parameters that progressively and quantitatively determine intubation difficulty, can be used to compare intubation difficulties in different situations [14]. The operator’s subjective assessment of intubation difficulty was evaluated using a numerical rating scale of 0–10 points.

The primary outcome was intubation time, and the secondary outcomes were the success rate of tracheal intubation in the first attempt, IDS score, operator’s subjective assessment of intubation difficulty, and modified Cormack–Lehane grades in the sniffing and ST positions.

### 2.3. Sample Size and Statistical Analysis

In a previous study, the standard deviation of tracheal intubation time was 15 s [15]. Assuming that a difference in intubation time between groups of >10 s is clinically meaningful, the number of participants calculated, with a power of 80% and α of 0.05, was 36 per group. Considering a 10% dropout rate, 120 patients (40 patients per group) were required.

Statistical tests were performed using R software (version 4.0.5; R Foundation for Statistical Computing, Vienna, Austria). Data were presented as mean ± standard deviation, median with interquartile range, or the number of patients. Categorical variables were analyzed using the Chi-squared test or Fisher’s exact test. Continuous variables were analyzed using one-way analysis of variance followed by Bonferroni’s post hoc test, or the Kruskal–Wallis test followed by the Mann–Whitney U test with Bonferroni correction, based on the normality test results. We used the Shapiro–Wilk test for normality testing of the variable distribution. The McNemar–Bowker test was used to determine the change in the modified Cormack–Lehane grades when changing from the supine sniffing position to the ST position in the ST direct and ST video groups. Statistical significance was set at *p <* 0.05. A corrected *p*-value < 0.017 (0.05/3) was adopted for intergroup comparisons.

## 3. Results

**Figure 2:**
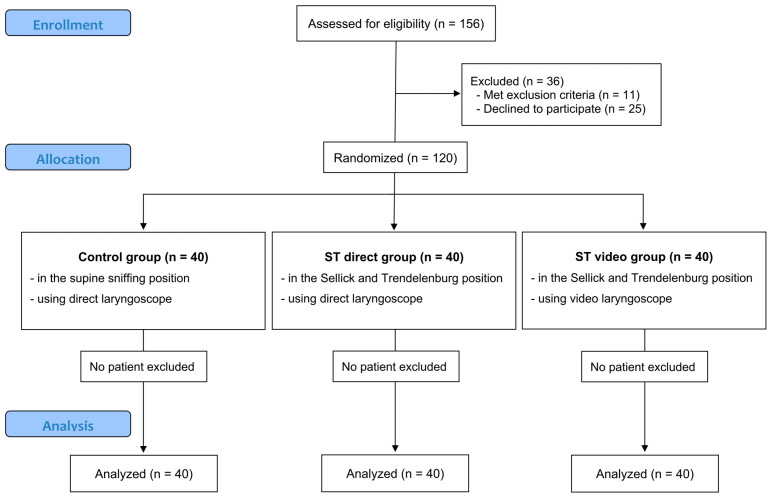
Consolidated Standards of Reporting Trials (CONSORT) flow chart of the study depicting the enrollment, randomization, and analysis.

A total of 120 of 156 eligible patients participated in the study after excluding 11 patients who met the exclusion criteria and 25 who did not consent to participate in the study. The patients were assigned to three groups, each comprising 40 patients (Figure 2); 54 were female and 66 were male. No statistically significant differences were observed between the groups regarding sex, age, height, weight, body mass index, ASA physical status, or Modified Cormack–Lehane grades in the supine sniffing position (*p* > 0.05; Table 1).

Table 2 presents the tracheal intubation time, first attempt success rate, and difficulty of tracheal intubation. The intubation times in the ST direct (*p* < 0.001) and ST video (*p* = 0.002) groups were longer than those in the control group. The first attempt success rate did not decrease in the ST video group (95.0%); however, it decreased in the ST direct group (77.5%, *p* = 0.007) compared with the control group (100%). Eleven cases of failed first attempt tracheal intubation (nine cases in the ST direct group, of which two were of modified Cormack–Lehane grade IV and the remaining were esophageal intubations, and two cases in the ST video group, which were esophageal intubations) were recorded. The second tracheal intubation attempt following the first failed attempt was successful. The difficulty of tracheal intubation was found to be higher in the ST direct group than in the other two groups based on the IDS scores (*p* < 0.001) and the operator’s subjective assessment of intubation difficulty (*p* < 0.001).

Table 3 shows the changes in the laryngoscopic view when changing from the supine sniffing position to the ST position in the two ST groups. No significant difference was observed in the modified Cormack–Lehane grades according to the position change in the ST video group; however, the modified Cormack–Lehane grades deteriorated significantly owing to the position change in the ST direct group.

The head-down tilt angle at which the imaginary line connecting the arytenoid cartilage and the corners of the mouth became horizontal in the ST position of the ST direct and ST video groups was 10.4 ± 3.5°.

## 4. Discussion

This study provides clinical data on tracheal intubation, examining the use of video laryngoscopes in the actual clinical application of the ST position to prevent pulmonary aspiration. Our study demonstrates that the difficulties associated with tracheal intubation when using the ST position can be mitigated by employing a video laryngoscope.

Although pulmonary aspiration is a rare perioperative event, serious respiratory complications are observed in nearly half of all cases, with a reported mortality rate of approximately 5% [1]. As most pulmonary aspiration incidents occur during anesthesia induction [16], appropriate efforts should be directed to prevent this complication when inducing anesthesia in patients at a high risk of pulmonary aspiration. Several methods, including the prevention of positive-pressure ventilation before intubation, cricoid pressure application, and appropriate patient position selection, have been incorporated to prevent pulmonary aspiration [3].

The advantages and limitations of patient positions, such as head-up, head-down, and supine, during anesthesia induction to prevent pulmonary aspiration have been debated. Although the head-up position has the advantage of suppressing passive regurgitation of the gastric contents [17,18], it cannot suppress active vomiting, which can lead to pulmonary aspiration [7]. The head-down position is preferred by some clinicians because the vomitus or regurgitated material does not fall into the airway, owing to gravity [19]. However, this position has the disadvantage of increased gastric pressure, which increases the risk of regurgitation, especially in obese patients [2,20]. The supine position is considered suitable for RSII; moreover, the risk of regurgitation and aspiration can be prevented by appropriate cricoid pressure application [21]. According to an international survey, <10% of anesthesiologists prefer the head-down position for RSII in current clinical practice [22]. This phenomenon can be attributed to concerns regarding the risk of gastroesophageal reflux or vomiting, as well as the perceived challenges associated with tracheal intubation, which outweigh the benefits of using the head-down position. In the Sellick position, the height of the corners of the mouth is similar to that of the carina; thus, the possibility of gastric contents being aspirated into the bronchi and lungs can be prevented even when gastric contents are regurgitated into the mouth. Additionally, the height of the corners of the mouth is similar to that of the larynx in the ST position, thus preventing the possibility of the aspiration of regurgitated gastric contents into the trachea [5,6]. The risk of increased gastric pressure and the patient sliding in the bed may arise in cases of the head-down tilt position wherein the patient’s oral cavity is lower than the larynx or carina [5,23]. Thus, the ST position appears to be more advantageous than the simple Trendelenburg (head-down) position.

The ST position may increase the difficulty of tracheal intubation due to the nonstraight path of the glottic opening from the mouth, unlike in the sniffing position. Additionally, difficulty may arise from the lack of familiarity or discomfort associated with using the ST position [6,8]. In a manikin simulation study by Pierre et al. [6], when intubation was performed with a head-down tilt of 15°, vocal cord visualization worsened and the mean intubation time was delayed by 10–15 s in the Sellick compared with sniffing position. Similarly, in this study conducted on actual patients, as the laryngoscopic view grade worsened, the difficulty of tracheal intubation increased, tracheal intubation time was delayed, and the first attempt success rate decreased in the ST direct group compared with the control group who underwent direct laryngoscopy in the supine sniffing position. By contrast, the tracheal intubation time was similarly delayed in the ST video group compared with the ST direct group; however, the difficulty of tracheal intubation and the first attempt success rate were not significantly different from those of the control group. Video laryngoscopes reportedly have certain advantages, such as a better view of the cords, higher intubation success rate, faster intubation, and fewer postoperative complications, over direct laryngoscopes in various situations [9,10,11]. Therefore, video laryngoscope in the ST position can be used in clinical settings as it can reduce the difficulty of tracheal intubation and increase the success rate, despite a slight delay in the intubation time.

Intubation in the ST position using a video laryngoscope is considered particularly useful for inducing anesthesia in patients with upper airway bleeding, such as tonsil, oral, or nasal bleeding, as well as vomiting or regurgitation of the gastric contents [2]. Bleeding in the upper airway impairs visibility around the glottis during tracheal intubation and increases the risk of aspiration of blood due to the pooling effect caused by gravity in the supine or head-up position. The use of the ST position is considered advantageous in this situation, and the use of a video laryngoscope may facilitate safe and easy tracheal intubation.

In some cases, upper airway bleeding and vomiting or regurgitation of the gastric contents may interfere with the view of the tracheal intubation, even in the ST position. However, even in the case of massive regurgitation, the concurrent use of a hard suction catheter reportedly helped maintain the visibility of the video laryngoscope and glottic visibility, allowing for successful intubation [24]. In our clinical experience, contamination of the video laryngoscope owing to blood or excessive secretions could be easily dealt with using suction, and we did not observe any significant difficulties when using the video laryngoscope in these situations. While more experience and research are still warranted, we believe that video laryngoscopy can still be considered a valuable tool in situations where regurgitation or upper airway bleeding is anticipated.

This study has several limitations that should be considered when interpreting the results. First, this study was not conducted in patients at high risk of pulmonary aspiration; thus, generalizability to these patients may be limited. However, since patients at risk of pulmonary aspiration do not have different airway anatomy, this is unlikely to make a significant difference. Additionally, in this study, manual ventilation was not avoided and higher doses of rocuronium were not administered as RSII was not required. Second, blinding of the operator performing tracheal intubation was not possible, which may have contributed to a bias. Third, this study was conducted only during tracheal intubation; therefore, data on the complications attributed to the ST position, such as neck pain, were not obtained. Fourth, the objective confirmation of appropriate neuromuscular blockade was not conducted as peripheral nerve stimulation monitoring was not utilized. Nevertheless, in all patients, airway assessment and intubation were performed at least 2 min after the intravenous administration of 0.6 mg/kg rocuronium [12], with no incidents of inadequate neuromuscular blockade reported. Fifth, we evaluated a single type of video laryngoscope with a curved blade, which is similar to a direct laryngoscope. Other types of video laryngoscopes, such as those with hyper-angled blades or specific intubation channels, may generate different results.

## 5. Conclusions

The use of a direct laryngoscope in the ST position complicates airway management by reducing the first attempt success rate of tracheal intubation. However, this challenge can be overcome by employing a video laryngoscope. Combining the ST position with a video laryngoscope offers a promising strategy to improve the success rate of tracheal intubation and prevent pulmonary aspiration. Future research should focus on evaluating specific types of video laryngoscopes or optimal stylet usage in the ST position and conducting dynamic simulations using manikins to further refine effective airway management strategies.

## Figures and Tables

**Table 1:** Patient characteristics.

Variables	Control Group (*n* = 40)	ST Direct Group (*n* = 40)	ST Video Group (*n* = 40)	*p*-Value
Sex (M/F)	26/14	19/21	21/19	0.268
Age	45.5 (38.8, 61.2)	42.5 (30.0, 54.0)	51.5 (40.8, 58.5)	0.289
Weight	71.3 ± 13.9	72.8 ± 13.9	69.1 ± 12.4	0.456
Height	169.5 (159.5, 175.2)	165.0 (160.2, 175.0)	168.0 (157.6, 173.0)	0.565
BMI	25.3 ± 3.5	26.1 ±3.9	25.4 ± 4.0	0.629
ASA I/II	26/14	28/12	29/11	0.761
Modified Cormack–Lehane grade using a direct laryngoscope in the supine sniffing position (I/IIa/IIb/III/IV)	13/10/10/7/0	17/10/8/5/0	15/12/9/4/0	0.930

Data are presented as mean ± standard deviation, number, or median and interquartile range. Control group: direct laryngoscopy in the supine sniffing position. ST, Sellick and Trendelenburg; M, male; F, female; ASA, American Society of Anesthesiologists; BMI, body mass index.

**Table 2:** Comparison of tracheal intubation time, first attempt success rate, and difficulty in each group.

Variables	Control Group (*n* = 40)	ST Direct Group (*n* = 40)	ST Video Group (*n* = 40)	*p*-Value
Intubation time (seconds)	28.0 (24.8, 34.0) *	36.0 (30.0, 48.0)	34.5 (29.8, 44.2)	<0.001
First attempt success rate	40/40 (100%)	31/40 (77.5%) ^†^	38/40 (95.0%)	0.002
IDS score	1.0 (0.0, 2.0)	2.0 (2.0, 3.0) *	1.0 (1.0, 2.0)	<0.001
Operator’s subjective difficulty evaluation (NRS)	1.0 (0.0, 3.0)	4.0 (2.0, 6.0) *	1.0 (0.0, 2.0)	<0.001

Data are presented as mean ± standard deviation, number (%), or median and interquartile range. Control group: direct laryngoscope in the supine sniffing position; Intubation time: the time from the insertion of the laryngoscope into the mouth to the measurement of the end-tidal carbon dioxide level (If the first attempt failed, the time taken for the first failed attempt was added to the intubation time), * *p* < 0.017 (0.05/3) compared with other two groups, ^†^ *p* < 0.017 (0.05/3) compared with the control group. ST, Sellick and Trendelenburg; NRS, numeric rating scale; IDS, intubation difficulty scale.

**Table 3:** Changes in the laryngoscopic view presented by the modified Cormack–Lehane grade when changing from the supine sniffing position to the ST position in the two ST groups.

Group	Supine Sniffing Position (I/IIa/IIb/III/IV)	ST Position (I/IIa/IIb/III/IV)	*p*-Value
ST direct group (*n* = 40)	17/10/8/5/0	1/5/14/17/2	<0.001
ST video group (*n* = 40)	15/12/9/4/0	10/16/10/3/0	0.791

Data are presented as numbers. The laryngoscopic view was evaluated with a direct laryngoscope in the supine sniffing position and with the laryngoscope of the relevant study group in the ST position. ST, Sellick and Trendelenburg.

## Data Availability

The datasets generated and analyzed in the present study are available from the corresponding author upon request.
